# AV block after flutter ablations?

**DOI:** 10.1007/s12471-014-0561-9

**Published:** 2014-05-15

**Authors:** A. W. G. J. Oomen, L. R. C. Dekker, A. Meijer

**Affiliations:** Catharina Hospital, Michelangelolaan 2, 5623 EJ Eindhoven, the Netherlands

## Rhythm puzzle-answer

**Fig. 3:**
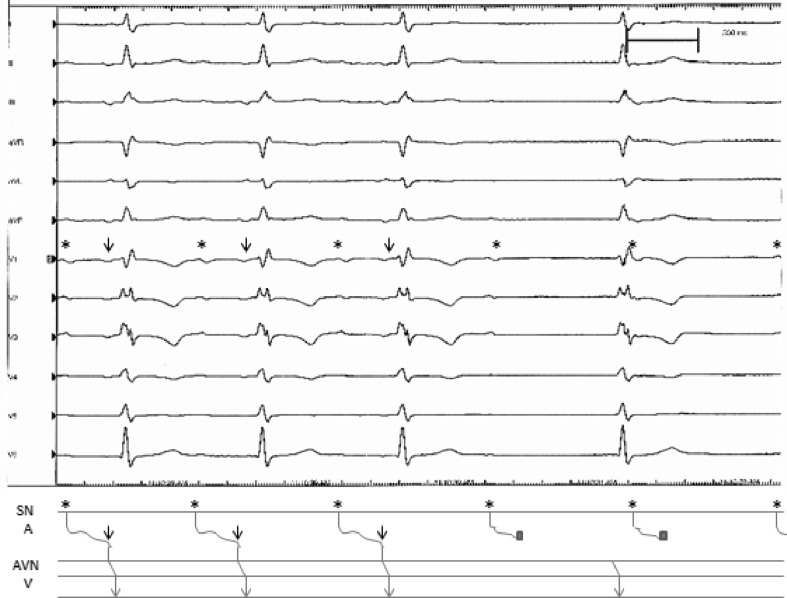
Rhythm recording including ladder diagram

The electrocardiogram in Fig. 1 shows a sinus rhythm of approximately 60 beats/min with two widely separated P-wave components. This reflects significant atrial conduction delay. The first three beats in Fig. 2 are similar to Fig. 1 with two distinct components, as shown by an asterisk and arrow in Fig. 3. However, the fourth P-wave only consists of the initial deflection (marked by an asterisk). There is intermittent atrial conduction block that causes the absence of the second component (indicated by arrows). Consequently there is no input to the AV node mimicking total AV block. This is also shown in the ladder diagram.

The atrial activation time at presentation was 300 ms versus only 90 ms before the last ablation. The sequence of events in this patient suggests that the last ablation in addition to the earlier ablation and heart surgery has caused significant inter-or intra-atrial conduction delay mimicking intermittent total AV block. This has rarely been documented (1, 2).

Input into the AV node was restored with implantation of a DDDR pacemaker. The atrial lead was placed posteriorly, low in the right atrial septum achieving intrinsic AV-nodal conduction to the ventricle. The patient was discharged home and is doing well up to 6 months later. Atrial conduction block remained present intermittently, yet less frequently.
